# Physical Simulation of Ultrasonic Imaging Logging Response

**DOI:** 10.3390/s22239422

**Published:** 2022-12-02

**Authors:** Junqiang Lu, Jiyong Han, Jinping Wu, Xiaohua Che, Wenxiao Qiao, Jiale Wang, Xu Chen

**Affiliations:** 1 State Key Laboratory of Petroleum Resources and Prospecting, China University of Petroleum (Beijing), Beijing 102249, China; 2 Key Laboratory of Earth Prospecting and Information Technology, Beijing 102249, China; 3 SINOPEC Engineering Technology Research Institute, Beijing 102200, China

**Keywords:** ultrasonic imaging, fracture response, physical simulation, amplitude

## Abstract

Ultrasonic imaging logging can visually identify the location, shape, dip angle and orientation of fractures and holes. The method has not been effectively applied in the field; one of the prime reasons is that the results of physical simulation experiments are insufficient. The physical simulation of fracture and hole response in the laboratory can provide a reference for the identification and evaluation of the underground geological structure. In this work, ultrasonic scanning experiments are conducted on a grooved sandstone plate and a simulated borehole and the influence of different fractures and holes on ultrasonic pulse echo is studied. Experimental results show that the combination of ultrasonic echo amplitude imaging and arrival time imaging can be used to identify the fracture location, width, depth and orientation, along with accurately calculating the fracture dip angle. The evaluated fracture parameters are similar to those in the physical simulation model. The identification accuracy of the ultrasonic measurement is related to the diameter of the radiation beam of the ultrasonic transducer. A single fracture with width larger than or equal to the radiation beam diameter of the ultrasonic transducer and multiple fractures with spacing longer than or equal to the radiation beam diameter can be effectively identified.

## 1. Introduction

With further oil exploration and exploitation, fractured reservoirs have become the focus of research and issues. In practice, the appearance of fractures has a great impact on oil production. At present, imaging logging is the most reliable fracture identification technology [1]. Compared with other imaging logging methods, ultrasonic measurement has the advantage of having no limitation on mud conductivity [2,3].

Ultrasonic imaging has been widely used in industrial inspections [4,5,6]. Zhang et al. [7] proposed a nondestructive evaluation method of coating thickness using water immersion ultrasonic testing. Accurately estimating ultrasonic time-of-propagation is essential in ultrasonic nondestructive testing (NDT). Lu et al. [8] estimated ultrasonic time-of-propagation using the echo signal envelope and modified the Gauss–Newton method. The nondestructive testing and evaluation (NDT&E) of internal defects is difficult due to the anisotropy and coarse grain of materials. Thus, a total focusing method for the ultrasonic annular array transducer was proposed and its imaging method was analyzed [9]. Given the increasingly stringent mandates from regulatory bodies and industrial leaders, the demand for ultrasonic transducers that can withstand harsh conditions has risen sharply and sensors for the ultrasonic NDT in harsh environments have been introduced [10,11].

Ultrasonic imaging logging was first researched in the 1960s. An ultrasonic imaging logging tool was originally designed to solve the problem of formation fracture evaluation [12,13,14]. In ultrasonic imaging logging, 2D imaging is carried out using the amplitude and arrival time of the reflected echo, which can comprehensively reflect the characteristics of the borehole wall. The imaging results can then be used to understand the borehole condition, conduct fracture detection, analyze formation [15], visualize the location of fractures and holes and effectively identify the shape of fractures. The inclination and orientation of the fracture can also be quickly calculated [16,17].

At present, ultrasonic imaging logging does not have an ideal geological application effect and cannot meet the requirements of exploration and exploitation of complex reservoirs. One of the important reasons for such a scenario is the unclear logging response to various influencing factors of ultrasonic imaging logging. The relationship between downhole typical geological characteristics and ultrasonic response has been established through model experiments, and the relationship between downhole geological body identification accuracy and the acoustic spot diameter of the acoustic beam radiated by the ultrasonic transducer is clarified. The findings provide reference and comparison for the development of ultrasonic imaging logging tools and the interpretation and geological application of logging data.

Logging while drilling (LWD) ultrasonic imaging logging has more complicated factors than wireline ultrasonic imaging logging. A physical simulation of the fracture response of ultrasonic imaging logging in the laboratory is necessary to provide reference and basis for the LWD ultrasonic imaging logging to evaluate the development of wellbore fractures. In this work, the experimental device and process are briefly introduced and ultrasonic imaging is carried out on slotted sandstone plate and fracture simulated borehole. The relationships of fracture width, fracture dip angle and hole diameter with ultrasonic echo amplitude and arrival time are also studied. This research provides a reference for the practical application of ultrasonic imaging logging (especially LWD ultrasonic imaging) in fracture identification and evaluation.

## 2. Experimental System and Methods

### 2.1. Experimental Equipment

**Figure 1:**
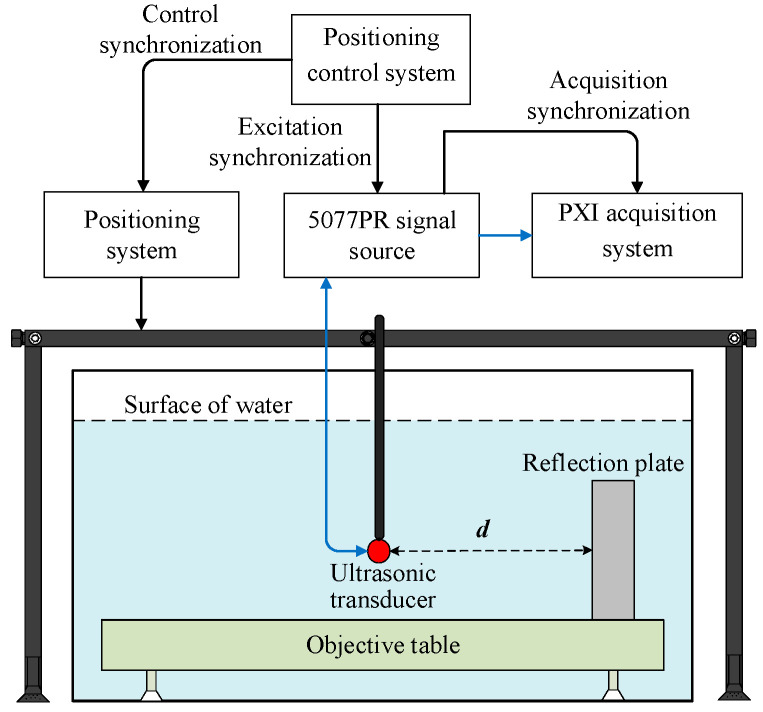
Schematic of the experimental equipment.

The schematic of the experimental equipment is shown in Figure 1. The experimental equipment includes the experimental measurement system, ultrasonic transducer and reflection plate.

#### 2.1.1. Experimental Measurement System

**Figure 2:**
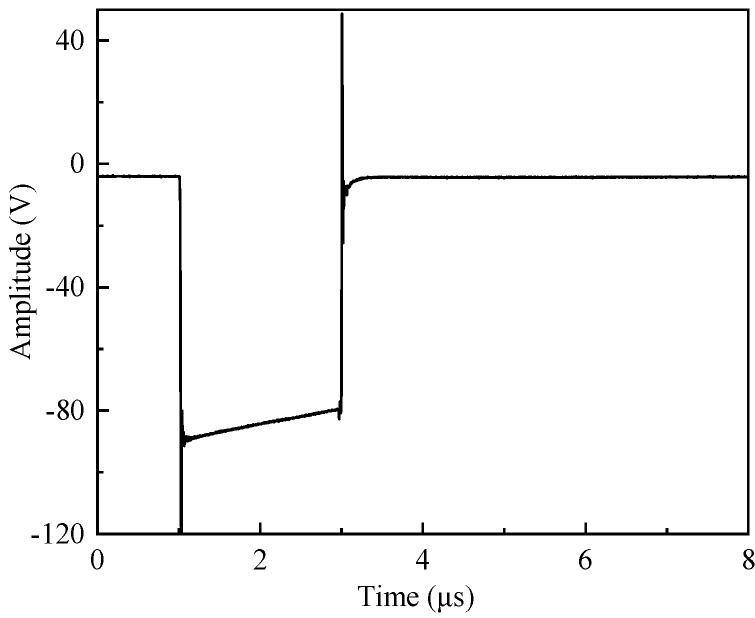
Time−domain waveform of the excitation signal.

The experimental measurement system was mainly composed of a high-precision positioning control system, 5077PR signal source, PXI acquisition system, objective table, ultrasonic transducer and water tank. The 5077PR signal source was used to generate a negative excitation pulse with amplitude of 100 V and a pulse width of 2 μs. The time-domain waveform of the excitation signal is shown in Figure 2. This signal drove the ultrasonic transducer in the experiments. The high-precision positioning control system directed the transducer to move according to the designed trajectory and consisted of control software and a positioning system to regulate the minimum moving step of the transducer to be 0.1 mm and the minimum rotating step to be 0.18°. The PXI acquisition system was used for real-time observation, acquisition and storage of experimental measurement waveform. The objective table was used to fix the reflection plate. The ultrasonic transducer transmitted acoustic signals and received reflected echoes.

#### 2.1.2. Ultrasonic Transducer and Its Radiated Sound Field Distribution

**Figure 3:**
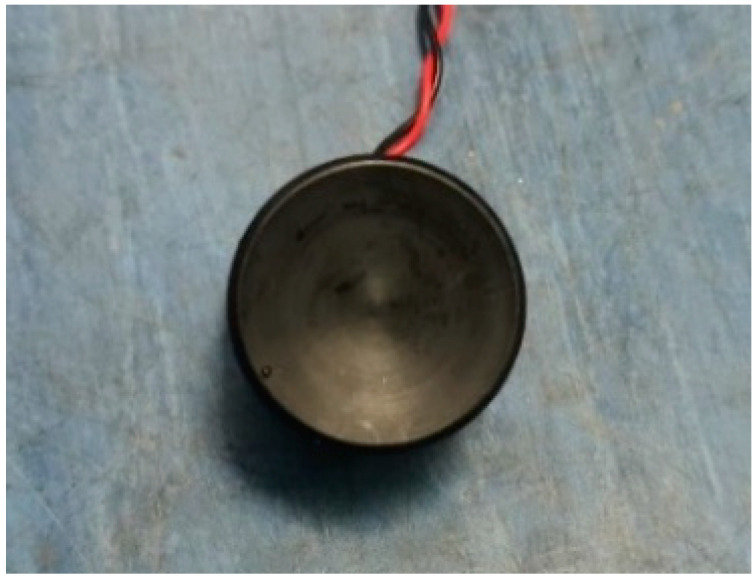
Photo of the ultrasonic transducer for physical simulation.

The actual photos of the concave ultrasonic transducer used in the experiment are shown in Figure 3. The transducer was used in the borehole LWD ultrasonic imaging tool. It had a radius of 15 mm, a concave curvature radius of 60 mm, a height of 20 mm and a dominant frequency of 250 kHz.

**Figure 4:**
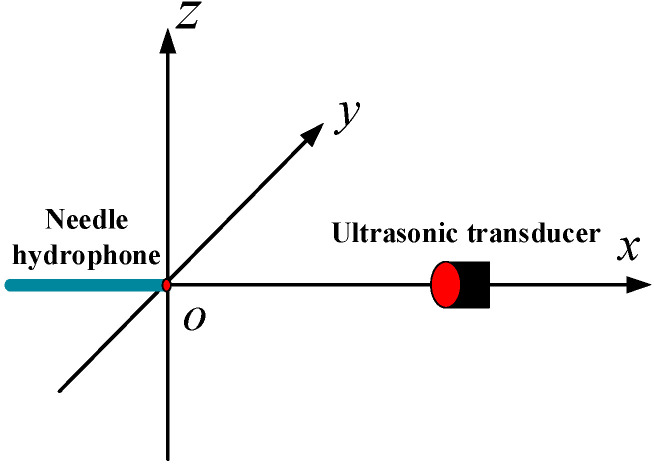
Schematic of the spatial distribution in the radiated acoustic field experiment.

**Figure 5:**
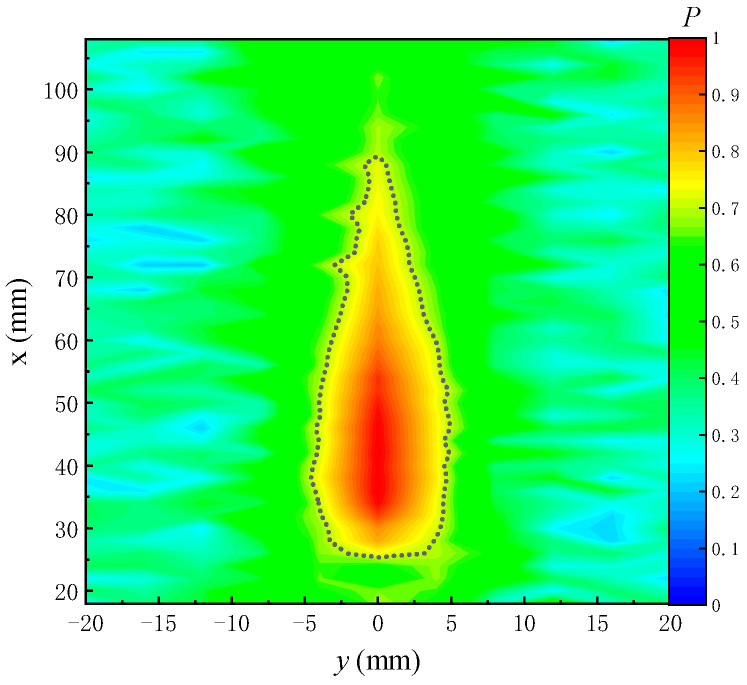
Spatial distribution of the radiated acoustic field perpendicular to the radiation surface of the ultrasonic transducer.

**Figure 6:**
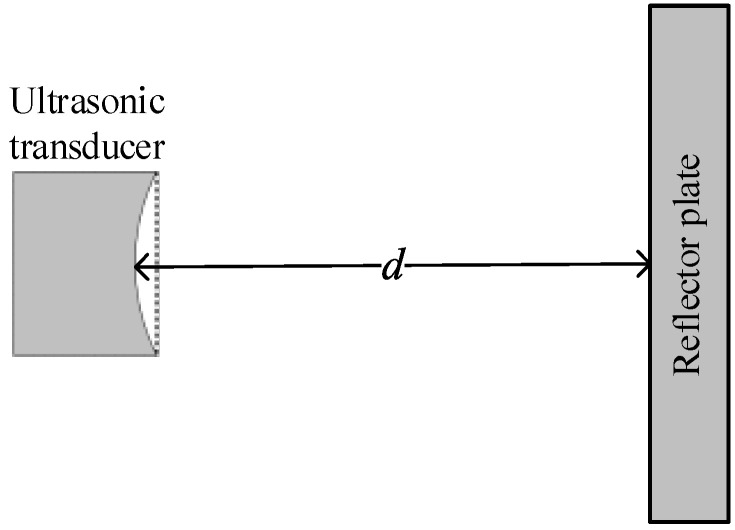
Diagram of distance d between the radiating surface of the ultrasonic transducer and the reflector plate.

Prior to testing the radiated sound field of the transducer, the spatial distribution of the radiated sound field of the ultrasonic transducer needs to be measured experimentally. The measurement scheme is shown in Figure 4. The normalized sound field distribution obtained by the experiment in the X−Y plane (z = 0 mm) is displayed in Figure 5, where the black dotted line is the contour with a value of 0.7. The range covered by the black dotted line was defined as the acoustic spot range of the transducer. In the following experiment, the distance *d* from the radiation surface of the transducer to the surface of the reflection plate (Figure 6) was 25–30 mm, and the width (sound spot diameter) in the Y direction was about 8 mm.

#### 2.1.3. Experimental Measurement Samples

(1) Plate reflector samples

**Figure 7:**
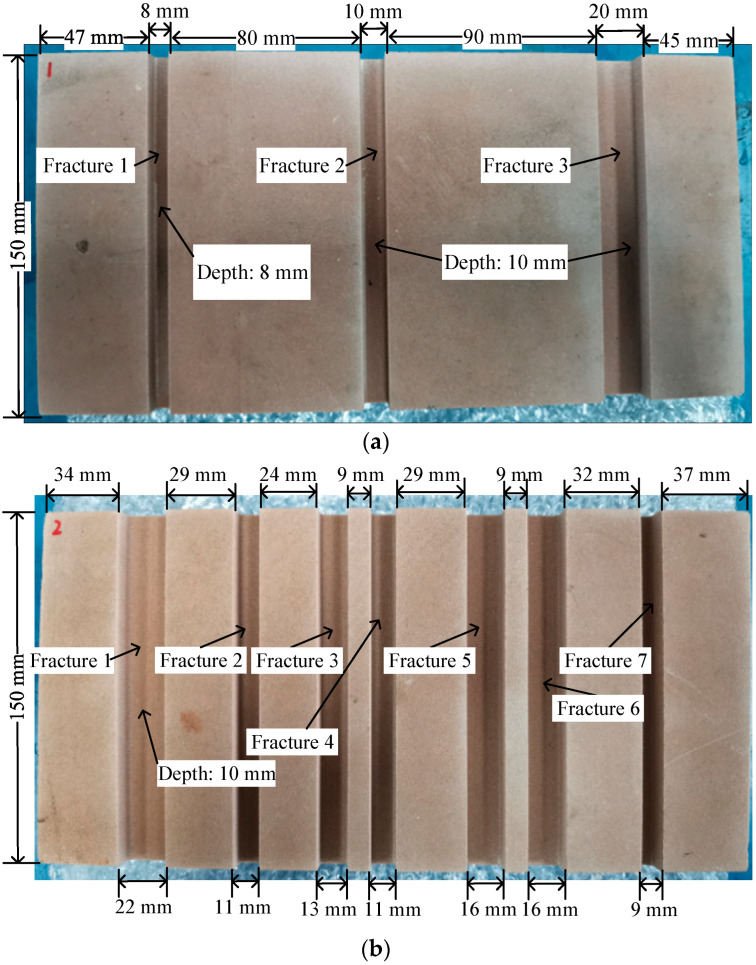
Reflector samples. (**a**) Grooved sandstone Plate 1 and (**b**) Grooved sandstone Plate 2.

As shown in Figure 7, the reflector samples are sandstone plates engraved with grooves of different widths and have a length of 300 mm, a width of 150 mm and a thickness of 48 mm. In the grooved sandstone Plate 1, the depth of Fracture 1 was 8 mm and that of Fractures 2 and 3 was 10 mm. In the grooved sandstone Plate 2, the depth of all fractures was 10 mm.

(2) Simulated borehole sample

**Figure 8:**
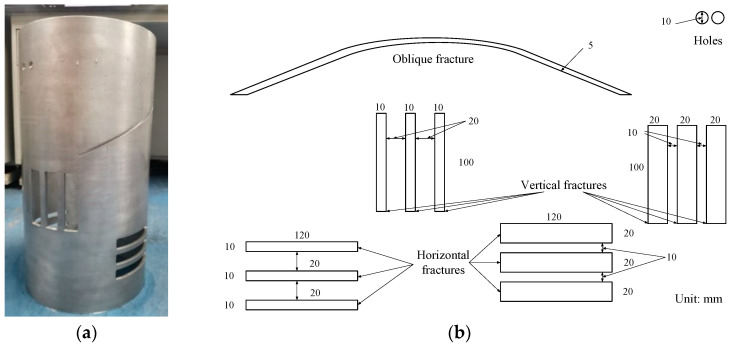
Fracture simulated borehole and its unfolded drawing. (**a**) Physical diagram of the simulated borehole and (**b**) Unfolded drawing of the borehole wall.

As shown in Figure 8a, an inner diameter of 216 mm, an outer diameter of 226 mm and a height of 500 mm was used for the aluminum cracked cylinder which was regarded as a simulated borehole. Figure 8b shows the unfolded drawing of the borehole wall and the specific parameters of the fractures. The simulated borehole exhibited holes, an oblique fracture and vertical and horizontal fractures.

### 2.2. Experimental Procedure

Ultrasonic imaging was carried out on slotted sandstone plates and fracture simulated borehole inner wall. The effects of fractures with different widths and shapes on the arrival time and amplitude of ultrasonic reflection echo were simulated and studied.

#### 2.2.1. Ultrasonic Imaging of Plate Reflector Samples

**Figure 9:**
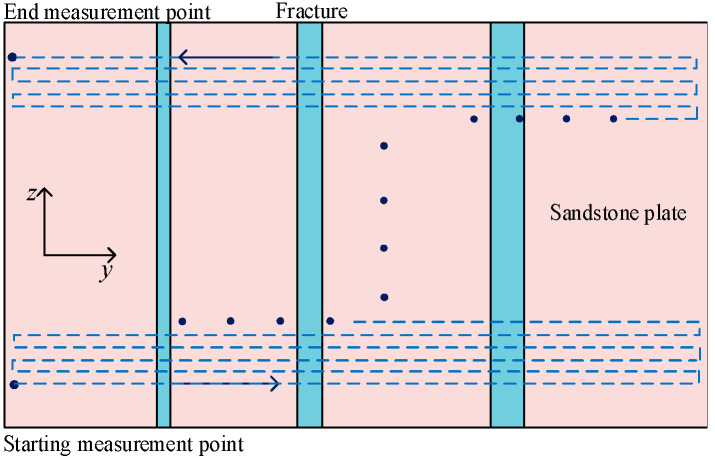
Schematic of the measurement method of slotted sandstone plate.

The distance *d* between the radiation surface of the transducer and the reflection plate was adjusted to 30 mm for simulating the distance between the radiation surface of the transducer and the borehole wall in the LWD environment. Before the grooved sandstone Plates 1 and 2 were measured experimentally, the transducer was moved to the starting measurement point of the model for measurement. In the experiment, the ultrasonic transducer started from the initial measurement point and scanned 270 times in the transverse direction (Y-axis) with a step size of 1 mm and scanned 50 times in the longitudinal direction (Z-axis) with a step size of 2 mm. The distance between the initial measurement point and the left boundary of the sandstone plate is 10 mm. The moving track of the ultrasonic transducer is shown as the dashed line in Figure 9.

#### 2.2.2. Ultrasonic Imaging of the Simulated Borehole

**Figure 10:**
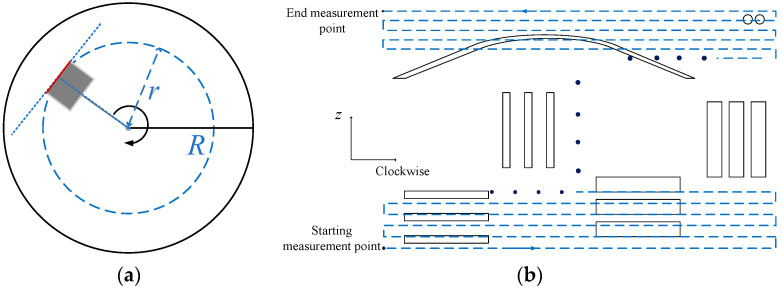
Schematic of the measurement method of the simulated borehole. (**a**) Schematic of the position of the transducer in the experimental measurement and (**b**) Schematic of the moving track of the ultrasonic transducer, unfolded drawing along the inner wall of the borehole.

For the scanning and measurement of the inner wall of a simulated borehole, the positional relationship between the transducer and the borehole wall is shown in Figure 10a. The moving track of the ultrasonic transducer is shown as the dashed line in Figure 10b and it is the unfolded drawing along the inner wall of the borehole. The borehole radius was 108 mm and the transducer radiation surface was fixed at a position of 83 mm away from the center of the rotation axis of the high-precision positioning system, to simulate the actual positional relationship between the transducer radiation surface and the borehole wall under the LWD condition. In scanning the inner wall of the simulated borehole, the ultrasonic transducer was first fixed at a depth point and then rotated clockwise for one circle with a step of 1.8°. Measurements were carried out at each step during the rotation. After each measurement, the ultrasonic transducer was moved upward (Z-axis) in steps of 2 mm and the circumferential measurement was then repeated. In the experiment, the ultrasonic transducer scanned 199 times in the circumferential direction with a step length of 1.8° and 194 times in the longitudinal direction with a step length of 2 mm.

## 3. Experimental Data Processing and Analysis

Ultrasonic imaging is carried out first on the plate reflector samples to study the influence of different width fractures on the ultrasonic pulse reflection echo and then on the inner wall of the simulated borehole sample to investigate the effect of different shapes of fractures and holes on the ultrasonic pulse reflection echo. Fracture inclination and hole size are also analyzed and calculated.

### 3.1. Experimental Results and Analysis of Plate Reflector Samples

**Figure 11:**
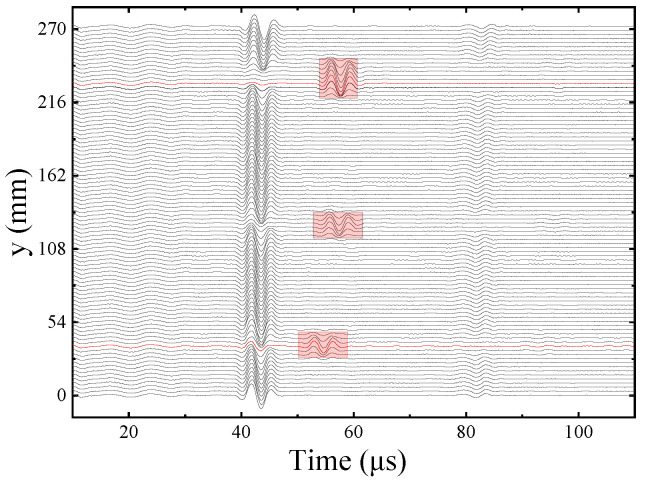
Measurement waveforms at the same height of grooved sandstone Plate 1.

**Figure 12:**
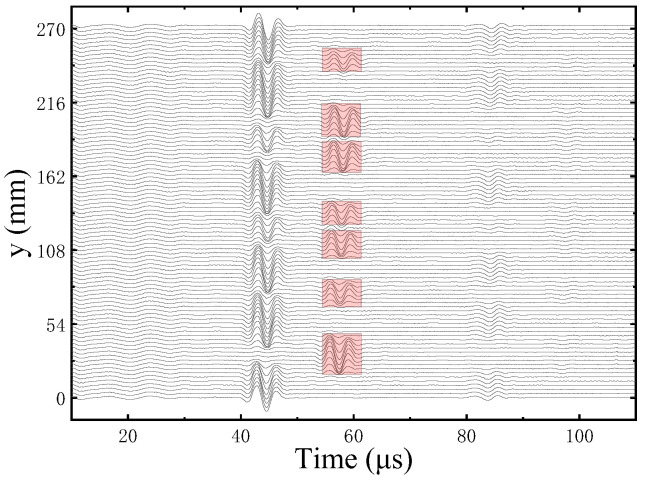
Measurement waveforms at the same height of grooved sandstone Plate 2.

Figure 11 and Figure 12 show all the waveforms measured at the same height point in the Z direction of the grooved sandstone Plates 1 and 2, respectively. The echo signals from the sandstone plate surface reached around 40 μs and those from the bottom of the grooved fractures reached around 50 μs (red rectangular box). These echo signals are consistent with the actual number of slotted fractures in the grooved sandstone Plates 1 (three grooved fractures) and 2 (seven slotted fractures).

**Figure 13:**
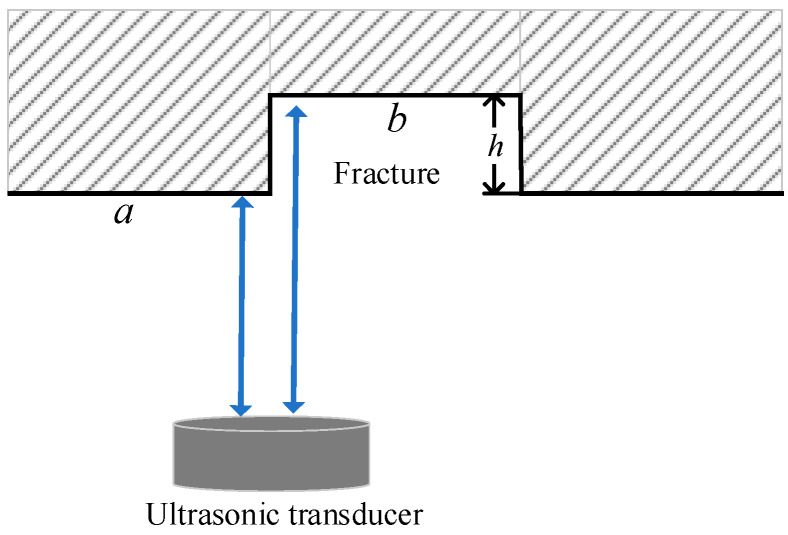
Schematic of the ultrasonic transducer scanning a fracture.

**Figure 14:**
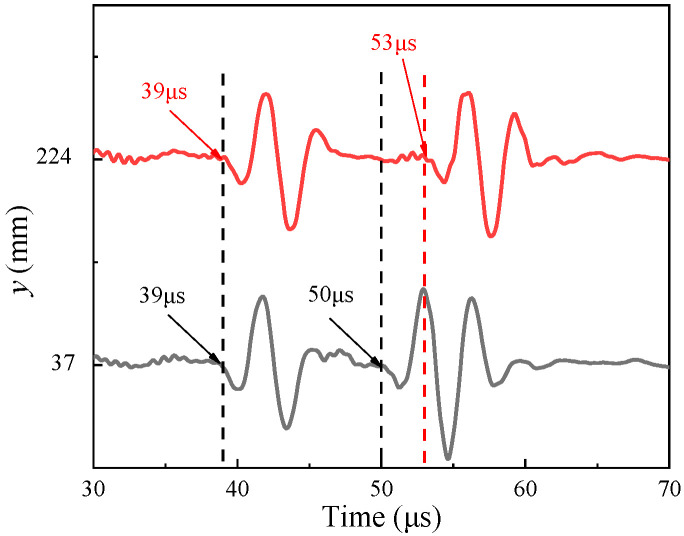
Arriving point extraction of reflected echo waveforms.

When the simulated fracture in Figure 13 was encountered during measurement, the ultrasonic transducer received the echo reflected by the two surfaces *a* and *b*, where surface *a* is the sample surface and *b* is the bottom surface of the groove. The dominant frequency of the ultrasonic transducer is 250 kHz, the longitudinal wave speed in water is 1500 m/s and the corresponding wavelength of the ultrasonic signal in water is about 6 mm. Given that the minimum depth of the grooved fracture of two sandstone plates is greater than a quarter of the wavelength, the depth of the grooved fracture can be calculated using the time difference of the reflected echoes from the two surfaces *a* and *b*. The reflected echo waveform and its arriving point extraction at two positions of sandstone Plate 1 are shown in Figure 14. The echo arrival time is extracted using the long and short window energy ratio method [18,19]. According to the echo signal arrival points in Figure 14, when y is 37 and 224 mm, the simulated fracture depth *h* is 8.25 and 10.5 mm, respectively, real fracture depth is 8 and 10 mm and the error is within 0.5 mm.

**Figure 15:**
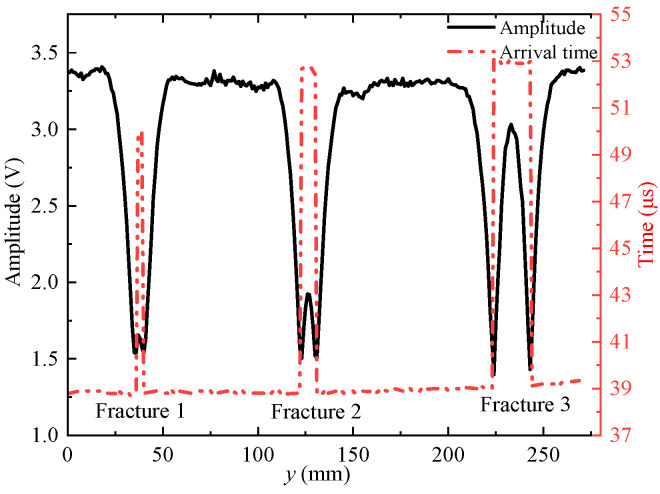
Echo amplitudes and arrival times for grooved sandstone Plate 1.

**Figure 16:**
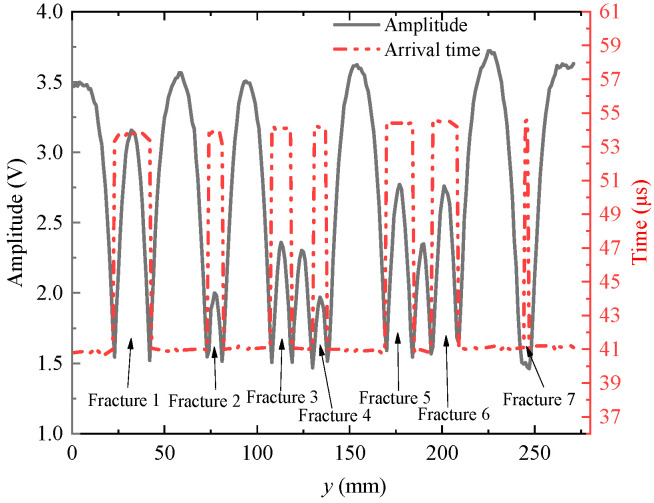
Echo amplitudes and arrival times for grooved sandstone Plate 2.

The echo arrival times of the waveforms are extracted by the long and short window energy ratio method and the echo amplitudes are extracted by the peak-to-peak method; the results are shown in Figure 15 and Figure 16. The amplitude of the reflected echo decreases at the position of the simulated fracture on the slotted sandstone plate and the arrival time of the reflected echo suddenly increases and then decreases at the position of the fracture. All waveforms measured on the grooved sandstone Plates 1 and 2 are windowed. The amplitude values are extracted by the peak-to-peak method and then imaged to obtain the 2D reflection echo amplitude imaging.

**Figure 17:**
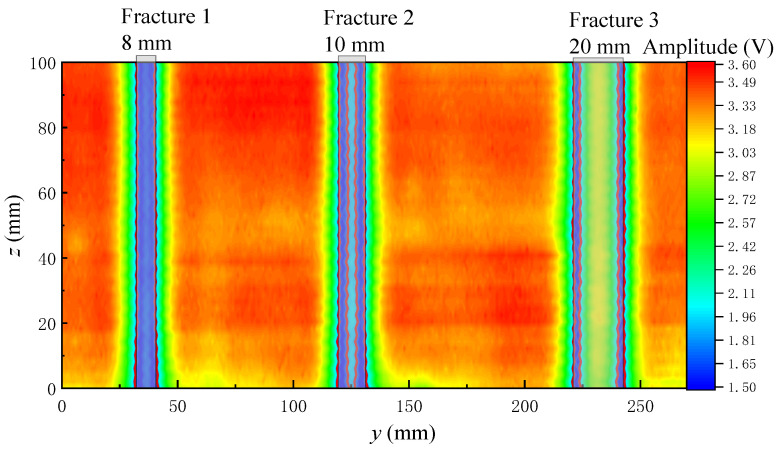
Echo amplitude imaging diagram for the grooved sandstone Plate 1.

**Figure 18:**
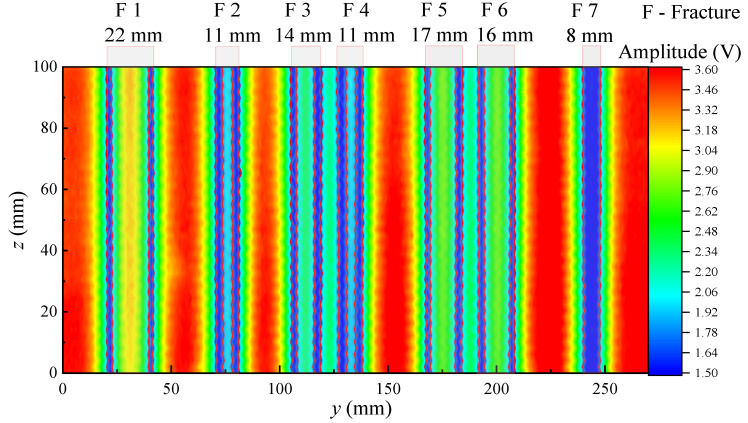
Echo amplitude imaging diagram for the grooved sandstone Plate 2.

Figure 17 and Figure 18 show the echo amplitude imaging of the grooved sandstone Plates 1 and 2. The boundary of the simulated fracture on the echo amplitude imaging map is evident and the echo amplitude drops remarkably. The position of the simulated fracture can be intuitively judged on the echo amplitude imaging map. The position where the echo amplitude decreases by half of the maximum value at the simulated fracture can be taken as the standard for identifying the boundary of the simulated fracture. The identification of the boundary position of the fracture helps to measure the width of the simulated fracture. Table 1 shows a comparison between the identified fracture width and the real value of the simulated fracture width.

When the ultrasonic transducer is used to scan and measure the simulated fractures with different widths at a distance of 30 mm from the grooved sandstone plate, the simulated fractures with a fracture width of 8 mm or larger can be identified (at this time, the fracture width is equal to the acoustic spot diameter of the ultrasonic transducer) with an error of within 2 mm and a relative error of within 12%.

The long and short time window energy ratio method is used to extract the arrival time of all the waveforms measured on the grooved sandstone Plates 1 and 2, and the arrival time is imaged to obtain the 2D reflection echo arrival time imaging.

**Figure 19:**
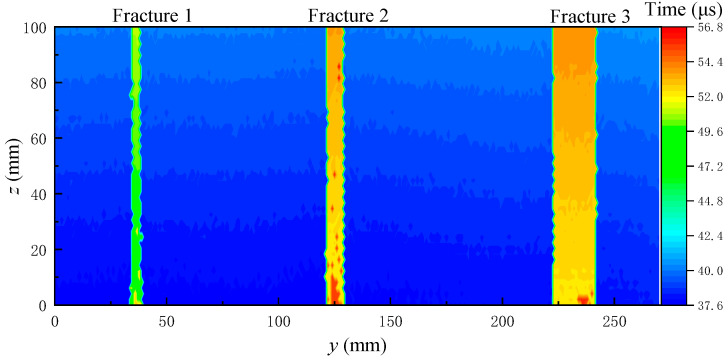
Echo arrival time imaging diagram for grooved sandstone Plate 1.

**Figure 20:**
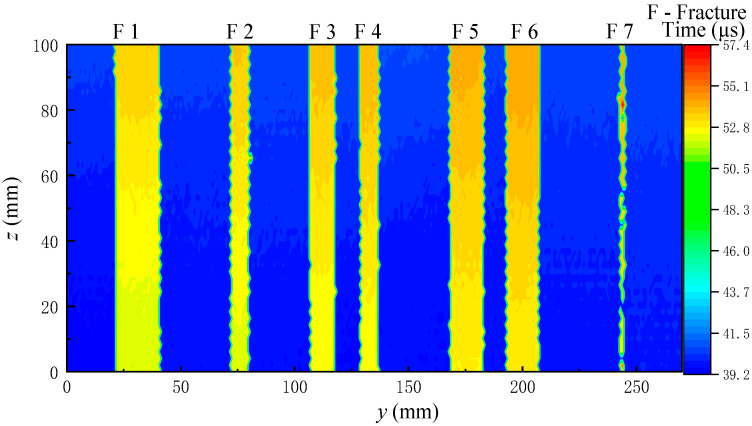
Echo arrival time imaging diagram for grooved sandstone Plate 2.

Figure 19 and Figure 20 show the echo arrival time imaging of the grooved sandstone Plates 1 and 2. The arrival time increases at the fracture on the slotted sandstone plate. Therefore, the position of the fracture can be identified by the echo arrival time imaging. When the fracture depth is different, the echo arrival time will also be different. For example, the echo arrival time varies between the narrowest Fracture 1 (depth of 8 mm) and other fractures (depth of 10 mm) of the sandstone Plate 1. When the distance between the two fractures is small and equal to the diameter of the acoustic spot radiated by the ultrasonic transducer on the reflection surface, (such as the 9 mm fracture spacing between Fractures 3 and 4 and between Fractures 5 and 6 in the sandstone Plate 2), the position of the fracture can be identified by imaging. Therefore, the fracture position and width can be identified by combining the echo arrival and amplitude imaging maps.

### 3.2. Experimental Results and Analysis of the Simulated Borehole

**Figure 21:**
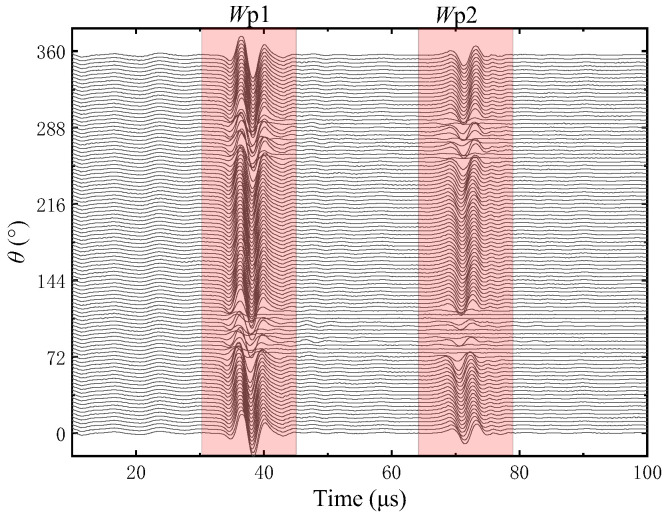
Circumferential echo waveforms for vertical fracture at the same height.

Figure 21 shows all the waveforms in the circumferential direction measured at the same height point in the Z direction of the vertical fracture of the simulated fracture development borehole. When the borehole wall has a fracture, the amplitude of the echo waveform received at the fracture position decreases and the vertical fracture in the simulated borehole is wider, which decreases more evidently. The red rectangular boxes on the waveforms are the primary and secondary echo wave packets reflected from the borehole wall, which are represented by Wp1 and Wp2, respectively. *θ* is the center angular corresponding to the circumferential direction of the simulated borehole.

**Figure 22:**
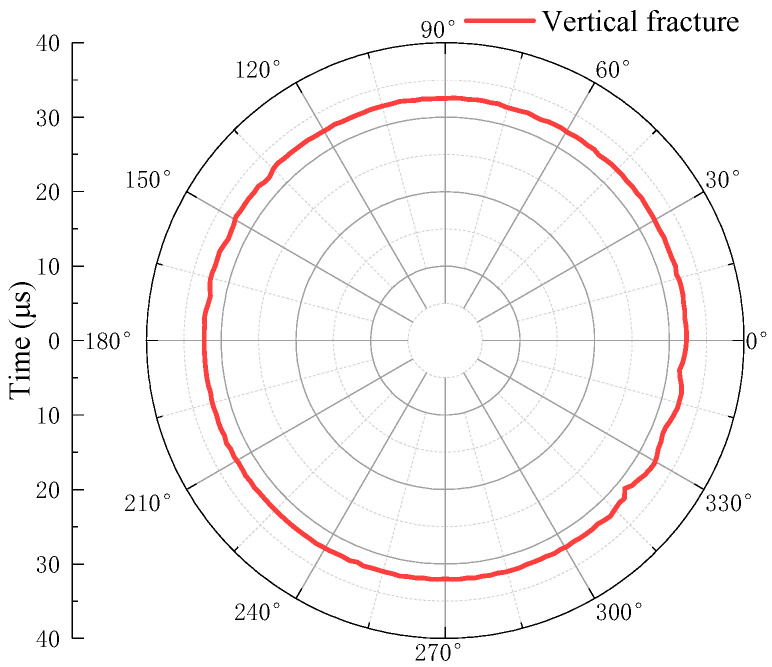
Echo arrival time of vertical fracture.

**Figure 23:**
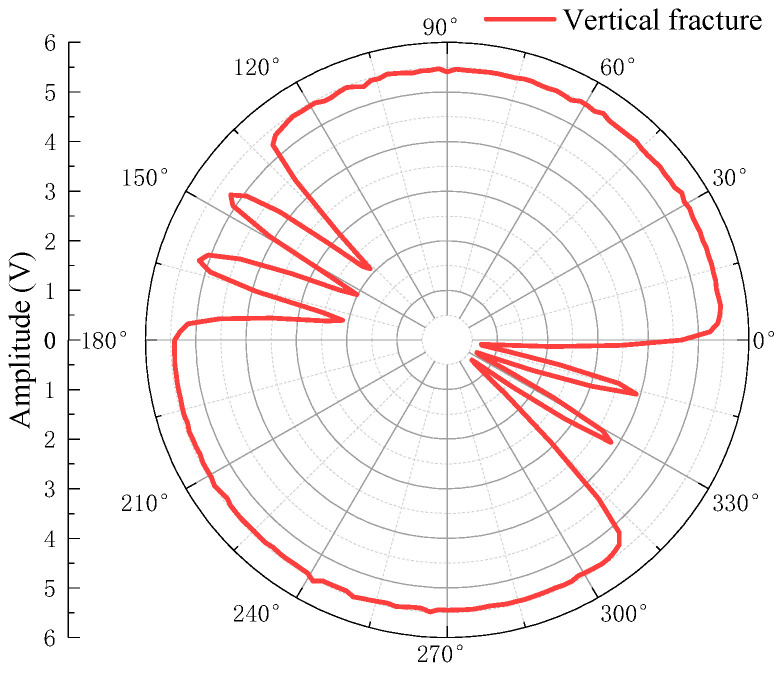
Echo amplitude of vertical fracture.

The waveforms are windowed and the arrival times of the primary echo Wp1 are extracted by the long and short window energy ratio method. The amplitudes of the primary echo Wp1 are then extracted via the peak-to-peak method. The arrival times and amplitudes of the echo Wp1 are shown in Figure 22 and Figure 23, respectively.

As shown in Figure 22, the echo arrival times of the borehole at this height point are similar, just as the arrival times of the scattered waves with small amplitude generated at the fracture edge are the same as those of the reflected waves on the wall surface without fracture. Hence, the fracture position is difficult to determine using the echo arrival time alone. A combination with the echo amplitude must be used for identification.

As shown in Figure 23, the borehole contains six fractures at this height point; the fracture width is the same at 130–180° and the fracture width is also the same at 310–360°. The fracture width of the former is larger than that of the latter. Thus, the echo amplitude at the simulated fracture decreases. The wider the fracture, the greater the amplitude reduction will be.

**Figure 24:**
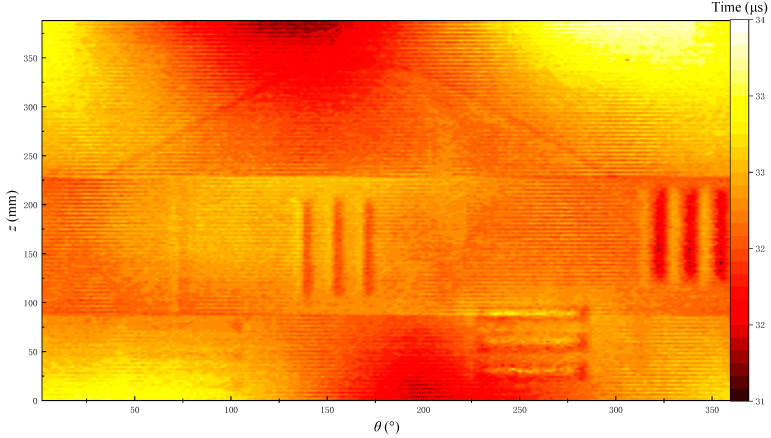
Echo arrival time imaging for the simulated borehole.

The echo arrival times of all the waveforms measured in the experiment are extracted through the energy ratio method of long and short time windows. The arrival times are imaged to obtain the 2D reflection echo arrival imaging as shown in Figure 24. Given that the fractures and holes on the simulated borehole are all hollow, the positions of the fractures and holes on the simulated borehole are difficult to distinguish based on the time arrival imaging. Therefore, the positions of fractures and holes must be analyzed through the echo amplitude imaging.

**Figure 25:**
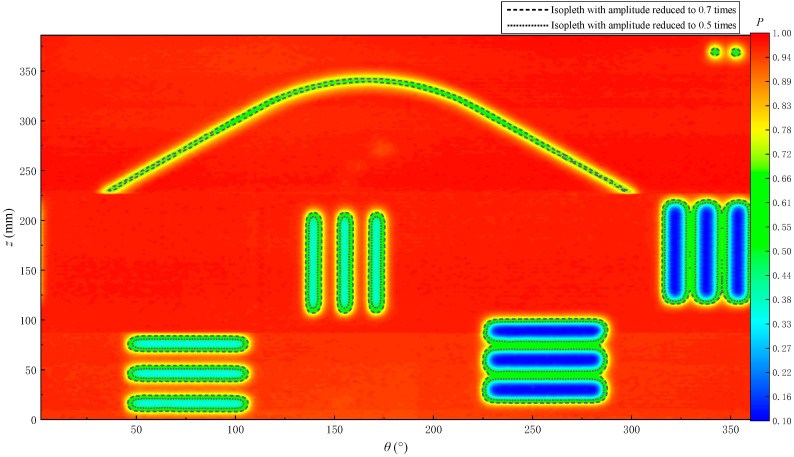
Echo amplitude imaging for the simulated borehole.

The amplitudes are extracted via the peak-to-peak method, normalized and then imaged. The 2D echo amplitude imaging is shown in Figure 25. The P in the figure represents the normalized sound pressure amplitude, which is dimensionless. The echo amplitude decreases at the positions of the fractures and holes in the simulated borehole, thereby visualizing the position of the fractures on the echo amplitude imaging. When the fracture width is smaller than the acoustic spot diameter of the ultrasonic transducer (8 mm), the echo amplitude at the fracture position exhibits less reduction. In Figure 25, the echo amplitude of the oblique fracture (5 mm wide) and the hole decreases to about 0.7 times that at the position without fractures (the black dashed line in Figure 25). When the fracture width is greater than or equal to the acoustic spot diameter of the ultrasonic transducer, the echo amplitude at the fracture position decreases remarkably. The larger the fracture width, the greater the amplitude decrease. For example, the amplitude of vertical and horizontal fractures in Figure 25 decreases to within 0.5 times the maximum value (the black dotted line in Figure 25).

**Figure 26:**
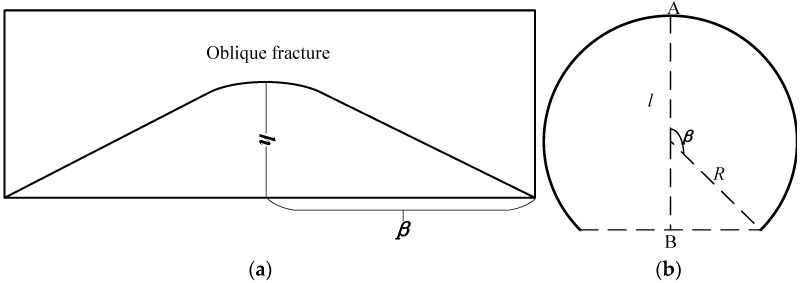
Schematic for the calculation of dip angle of oblique fracture. (**a**) Side expanded view of oblique fracture. (**b**) Projection of oblique fracture on the horizontal plane.

The dip angle of the oblique fracture in the simulated borehole is calculated, and the side expanded view and its projection on the horizontal plane is shown in Figure 26. *R* is the borehole radius, point *A* is the highest point of the fracture, point *B* is the midpoint of the line connecting between the two lowest points of the fracture and *h* is the distance between points AB in the side expanded view. *l* is the distance between points AB, *β* is the center angle of the arc formed between point A and the lowest point in the projection on the horizontal plane. The formula to calculate the oblique fracture dip angle *α* is as follows [20]:

(1) $$\alpha =\arctan\left(\frac{h}{l}\right)$$

The calculation formula of *l* is as follows:

(2) l=R[1+cos(180°−β)]

**Figure 27:**
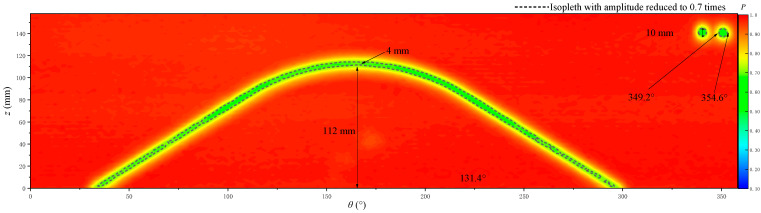
Echo amplitude imaging of oblique fractures and holes.

The echo amplitude imaging of oblique fractures and holes is shown in Figure 27. *h* is 112 mm, *β* is 131.4°, the fracture dip *α* calculated from Equations (1) and (2) is 31.96°, the actual value of the fracture dip is 30° and the difference between the calculated value and actual value is 6.5%. These values indicate that the identification result of the fracture dip is reliable. The width of the oblique fracture obtained on the imaging map is about 4 mm, which is 20% lower than the real value of the simulated fracture width at 5 mm. The main reasons for errors are as follows. Given that the amplitude change between adjacent data points on the imaging map is a gradual process, errors may have arisen during the fracture boundary identification. In addition, the setting of the measurement step can cause measurement error at the fracture boundary. For example, the step length in the z direction is 2 mm, so the fracture width identification result is most reliable when the number is even. Some errors might have occurred when using the true value of the oblique fracture of 5 mm. However, the echo-response near the fracture edge is gradual. The width of the oblique fracture is 4–6 mm and its response on the imaging map is evident.

The hole position and size can also be identified through the amplitude imaging. The hole diameter along the z direction and the angle range of the borehole circumferential expanded view corresponding to the hole diameter are marked in Figure 27. The arc length formula can be used to calculate the arc length of the hole in the borehole circumferential direction so that the hole diameter can be obtained. The arc length formula is as follows:

(3) $$k=\frac{n\pi R}{180}$$

where *k* is the arc length, *n* is the central angle corresponding to the arc length and *R* is the borehole radius.

*k* is calculated to be 10.17 mm and the difference between the calculated hole diameter and actual hole diameter of 10 mm is 1.7%, thereby indicating that the experimental method is effective in hole identification.

## 4. Conclusions

Most of the physical simulation experiments of ultrasonic imaging logging have analyzed the influence of transducer frequency, shape of transducer radiation surface, scanning parameters and measurement environment on the measurement results. In this work, the ultrasonic imaging logging response of typical geological features is experimentally studied. Ultrasonic imaging is carried out on the grooved sandstone plates and simulated boreholes and the ultrasonic reflection echo of the grooved sandstone plates and simulated boreholes with fractures or holes of different widths and shapes is measured. The amplitude and arrival time of the reflected echo are analyzed. According to the experimental results, the following conclusions can be obtained:

(1) When the ultrasonic transducer is at the fracture boundary and the fracture depth is greater than a quarter of the ultrasonic signal wavelength, two wave packets will appear in the reflected echo waveform. The arrival times of two wave packets are extracted and imaged and the depth of the fracture can be determined.
(2) The fracture identification accuracy of the ultrasonic imaging logging is related to the acoustic spot diameter radiated by the ultrasonic transducer on the reflecting surface. A fracture with a width equal to or greater than 8 mm of the ultrasonic transducer’s sound spot diameter and multiple fractures with a fracture spacing greater than or equal to the sound spot diameter can be effectively identified.
(3) When the simulated borehole is measured, the distance between the transducer and reflective surface is small and the diameter of the acoustic spot is reduced. Fractures with a width of 5 mm can be effectively detected. Large fracture depths and great attenuation of the echo wave at the fracture are conducive to the identification of fractures.
(4) The position and scale of fractures and holes can be identified by combining the echo amplitude imaging and arrival time imaging. If the fracture and hole are small, then the arrival time of the scattered wave from the edge is basically consistent with that of the reflected wave from the surface without fractures and holes. Thus, the arrival time of the echo wave alone cannot be used to identify the fracture and must be combined with the echo amplitude.

## Figures and Tables

**Table 1:** Comparison of identified fracture width with real values.

	Fracture No	Identified (mm) Width/mm	Real (mm) Width/mm	Error (mm)	Relative Error (%)
Grooved sandstone Plate 1	Fracture 1	8	8	0	0
	Fracture 2	10	10	0	0
	Fracture 3	22	20	2	10
Grooved sandstone Plate 2	Fracture 1	22	21	1	4.7
	Fracture 2	11	11	0	0
	Fracture 3	14	13	1	7.7
	Fracture 4	11	11	0	0
	Fracture 5	17	16	1	6.3
	Fracture 6	16	16	0	0
	Fracture 7	8	9	1	11.1

## Data Availability

The data presented in this study are available upon request from the corresponding author.
